# Identification of the effects of acid-resistant *Lactobacillus casei *metallopeptidase gene under colon-specific promoter on the colorectal and breast cancer cell lines

**DOI:** 10.22038/ijbms.2021.53015.11950

**Published:** 2021-04

**Authors:** Narges Dadfarma, Jamileh Nowroozi, Bahram Kazemi, Mojgan Bandehpour

**Affiliations:** 1 Department of Microbiology, Faculty of Biological Sciences, North Tehran Branch, Islamic Azad University, Tehran, Iran; 2 Cellular and Molecular Biology Research Center, Shahid Beheshti University of Medical Sciences, Tehran, Iran; 3 Department of Medical Biotechnology, School of Advanced Technologies in Medicine, Shahid Beheshti University of Medical Sciences, Tehran, Iran

**Keywords:** Apoptosis, Cytotoxicity, Lactobacillus casei, Recombinant plasmid, TP53 and MAP2K1 genes - expression

## Abstract

**Objective(s):**

Anti-tumor effects of Lactobacilli as normal flora have been described. In a previous study, we identified a protein isolated from the bacterium *Lactobacillus casei* ATCC 39392 in acidic pH conditions named metallopeptidase. Therefore, we decided to evaluate the effect of the recombinant plasmid coding metallopeptidase protein on the inhibition, proliferation, or apoptosis of the colorectal and breast cancer cell lines.

**Materials and Methods:**

Identified metallopeptidase gene of *L. casei* under the specific colon cancer promoter was transferred to the Human SW480 and MDA-MB231 cells. Cell viability was evaluated in these two cancer cell lines via MTT assay, apoptotic changes, and expression level of p53 and *MAP2K1* genes in comparison with healthy blood cells as a control group.

**Results:**

Viability of SW480 and MDA-MB231 cells was identified at 25% and 7%, respectively. An increase in apoptotic cell death in the SW480 cell line was observed as revealed by Tunnel staining. The expression assay of *TP53* and *MAP2K1* genes showed that MPL protein altered gene expression in a cell type-specific manner. Tunnel analyses showed that the pronounced cytotoxic effect of pEGFP-C2/MPL plasmid on SW480 cells was mediated through apoptosis.

**Conclusion:**

These results suggest that endogenous recombinant MPL under colon specific promoter inhibits the proliferation of SW480 colorectal cancer cells by increase in MAP2K1 and P53 activation. *L. casei* metallopeptidase under the same circumstances could not affect the growth rate and viability of MDA-MB231 breast cancer cells *in vitro*.

## Introduction

Colorectal cancer (CRC) is the third most common form of cancer (1). There is no specific way to identify the early stages of colon cancer, and advanced cancer treatment is by chemotherapy which has severe side effects and systemic toxicity because these drugs attack all dividing and replicating cells. Thus, there is a continuously growing demand for a novel anti-cancer agent (2). Breast cancer is another common malignancy, accounting for approximately one-third of all cancers occurring in women (3). According to the report of GLOBOCAN, the Global Cancer Observatory (2018), breast cancer incidence, mortality, and 5-year (2014–2018) prevalence were estimated to be the highest for women, whereas mortality and 5-year (2014–2018) prevalence for colorectal cancer incidence were ranked second and third for women and men, respectively (4). Therefore, various studies are being conducted to improve therapeutic approaches, including the development of effective biomarkers for CRC detection, the identification of biotherapeutic drugs with fewer side effects, including antibodies and therapeutic proteins and enzymes whose clinical effects have been proven in humans (5, 6). Considering the toxic side effects of chemotherapy in the treatment of cancer, anticancer drugs with minimal or no side effects of natural origin including probiotic *Lactobacillus* strains have recently received more attention. 

The lactic acid bacteria (LAB), are a group of gram-positive, non-spore forming, fermentative, catalase-negative, non-motile microorganisms. Some LAB strains are well known for their probiotic properties (7). They are conventionally used as antimicrobial, antidiabetic, immunomodulatory, bio preservative, immune system booster, and gut microflora (8). LAB may suppress the growth of bacteria that convert procarcinogens into carcinogens, thereby reducing the number of carcinogens in the human intestine. They also produce short-chain fatty acids in the colon, which acidify the environment. They can influence bacterial enzyme activity related to the production of carcinogenic compounds, such as beta-glucuronidase, nitroreductase, and azoreductase. Some of these beneficial effects may be attributed to secreted probiotic-derived factors, like soluble metabolites. Such metabolites have recently been identified as “postbiotic” mediators (9) which are secreted and released into the environment by probiotic bacteria and participate in the interaction between symbiotic bacteria with the host epithelial and immune cells (10-12). According to Sanchez *et al*. findings, the commensal microbiota and the host cells have a continuous exchange of molecular information called cross-talk. Very little is known about the cellular receptors responsible for the recognition of extracellular proteins secreted by probiotic bacteria (11). Most of the characterized probiotic–host interactions take place through pattern recognition receptors (e.g., Toll-like receptors, TLRs) which recognize common molecules present on the bacterial surface (peptidoglycan and lipoteichoic acids) or derived from them (e.g., unmethylated CpG DNA and exopolysaccharides). After binding, components of probiotics like MAMPs to PRRs regulate nuclear factor kappa B (NF-κB), mitogen-activated protein kinases (MAPK), peroxisome proliferator-activated receptor gamma, and other signaling pathways (10,13). In addition, some metabolites produced by probiotics, such as secreted proteins (extracellular proteins), organic acids, indole, bacteriocins, extracellular vesicles, H2O2, and NO, protect the gut’s epithelial barrier by boosting mucus secretion by goblet cells, increasing the production of antimicrobial peptides, or enhancing the expression of tight junctions (14).

Probiotics change key signaling pathways in intestinal epithelial cells. Thereby the key biological signaling pathways like NFκB, MAPK, Akt/PI3K, and PPARγ are targets for probiotics or their products (15). Previous studies have reported that different species of the genus *Lactobacillus *could inhibit colon cancer progression, however the exact molecules involved have not yet been identified (2). *Lactobacillus casei* ATCC 39392 is a key microorganism in fermented dairy products and foods. The anti-proliferative effects of this strain have been demonstrated on several cancer cell lines, including human (HT-29) and mouse (CT26) colon cancer cell lines (9). Supernatants and bacterial extracts of this standard bacterium were treated in CaCo-2 cells (16). The effect of growth inhibition of colon carcinoma in BALB/c mice with oral administration of *L. casei* ATCC 39392 (2) and liver cancer Huh7 cells (17) have been reported. The beneﬁcial eﬀects of *Lactobacilli* secreted products that have been exerted through mechanisms similar to those described for probiotics include (1) competition with pathogens for binding to the receptors, nutrients, and colonization; (2) promotion of intestinal epithelial cell survival and barrier function; (3) stimulation of innate immunity and reduction of pathogen-induced inﬂammatory bowel diseases (18). Some of them are produced in the supernatant, while some are also structural components of the bacterium. The supernatants are not involved in the growth, proliferation, and evolution of bacteria, and are usually produced in the stationary phase of the growth of bacteria. Since there are a number of inherent problems in application of direct live bacteria, it seems using probiotic byproducts would be more beneficial and safer (19). Many studies on human mucosa organ culture have shown that some probiotics are detrimental to inflammatory bowel diseases. The study of extracellular proteins may provide novel strategies for clinical application of probiotic bacteria and may allow understanding of their mechanism of action and interaction between probiotic bacteria and the human host cells. Extracellular proteins secreted by probiotic lactobacilli have been shown to help maintain the mucosal barrier, mainly through MAPK-dependent mechanisms (11). Two protein produced by LGG, p40 and p75, have been shown to promote IEC homeostasis (6, 8, 12).

Based on Chull reports, a novel therapeutic probiotic-derived protein, P8, with anti-colorectal cancer (anti-CRC) properties in CRC cell line (DLD-1), had low penetrative efficiency, and they tried to improve delivery to CRC cells and expressed P8 as endogenous in order to improve P8 therapeutic efficacy, which doubled its anti-proliferative activity. They showed that endogenous P8 expression suppresses growth of CRC cells by inducing cell cycle arrest through inhibiting Cdk1/Cyclin B1 activation via the p53-p21 pathway (5). Probiotics encounter extreme environmental conditions during food processing or along the gastrointestinal tract. In most cases, multiple biological functions are affected upon exposure of the cell to environmental stress. Sensing of sublethal environmental stress can allow for adaptation processes to occur, which can include alterations in the expression of specific proteins (20).

 In some studies, the responses of various probiotics against different environmental stresses such as acid stress have been studied by comparative proteomic analysis to identify proteins important in acid tolerance and other environmental stress conditions (20-24).

Similar to this type of research on therapeutic effects of this microbe, we examined the effects of acid stress on the intracellular proteome of *L. casei *ATCC 39392 and using proteomics methods we identified a metallopeptidase protein (25).

However, secretion and cell wall anchoring of proteins are key mechanisms of LAB interaction with their environment. Optimization of protein export is valuable when using these food-grade microorganisms for biotechnological applications both *in vitro *and *in vivo *in humans and animals, and for development of recombinant LAB as a live, vaccine-delivery vehicle (7).

In the present study, a gene putatively encoding a metallopeptidase protein of *L. casei* ATCC 39392 was cloned and characterized under specific colon cancer cell promoter. The anti-proliferative and apoptotic activities of the endogenously expressed metallopeptidase protein were evaluated on SW480 and MDA-MB231 cell lines and compared with WBC blood cells as control.

## Materials and Methods

***Metallopeptidase gene construction***

The sequence of metallopeptidase protein conducted

from mass spectroscopy of isolated protein in *L. casei *ATCC 39392 cultivation in acidic condition was consiered for plasmid design. The peptide sequences of secreted protein were compared with protein sequences in the NCBI microbial blast (https://blast.ncbi.nlm.nih.gov/Blast.cgi?PAGE_TYPE=BlastSearch&BLAST_SPEC=MicrobialGenomes) using BLAST analysis. The sequence was retrieved from Uniprot (https://www.uniprot.org/). After reverse translation of protein sequence (https://web.expasy.org/translate/), it was codon-optimized for human cell culture by JCAT online server (http://www.jcat.de/) and named MPL. The colon cancer cell-specific promoter (GCTTCCGCCCACGGCTTCCCCTCCCCCTCGCCGACGGCCCAAGGCGTTCAGGCACGCGCTATGGCGCTGTGGATGCGCCTGCTGCCGCTGCTGGCGCTGCTGGCGCTGTGGGGCCCGGATCCGGCGGCGGCG) was obtained from promoter database (https://epd.epfl.ch//index.php) which was inserted upstream of the metallopeptidase gene. This sequence was subsequently cloned into the pEGFP-C2 eukaryotic expression vector between *EcoR1* and *Xho*I restriction sites with a hexahistidine tag at N-terminus and named pEGFP-C2/MPL plasmid. The pEGFP-C2 expression vector was used as a delivery vehicle

for endogenous expression of MPL protein. Restriction

striction analysis was applied for the confirmation of the

synthesized plasmid. 

***Cell lines, growth conditions, and transfection***

Human SW480 (ATCC® CCL-228™) and MDA-MB 231 (ATCC® HTB-26™) cell lines were purchased from the Pasteur Institute of Iran. They were cultured in standard DMEM (GIBCO, USA) medium supplemented with 10% fetal bovine serum (FBS) (GIBCO, USA), 2 mM glutamine, 100 µ/ml penicillin, and 100 μg/ml streptomycin (GIBCO, USA) at 37 °C in a humidified atmosphere of 5% CO_2_. The cells were washed twice with PBS, harvested with 0.05% trypsin, and transferred (10^6^ cells/ml) into a 24-well multi-dish (100 µl/well) containing serum-free fresh medium without antibiotics. The microplates were kept at 37 °C in 5% CO_2_ until cell lines formed a monolayer in each well. We have used Lipofectamine® 3000 Reagent (Invitrogen) for transfection and delivered 10 µg plasmid per well containing SW480, MDA-MB 231, and blood WBC cell culture as control. The WBCs were collected using Ficoll® Paque Plus according to the manufacturer’s instructions. 

***GFP expression***

For evaluation of colon cancer specific promoter function, we inserted the MPL gene under promoter in upstream of the GFP gene in MPL construct. So, after 24 hr from transfection of all of the cells by pEGFP-C2/MPL plasmid, they were studied by fluorescent invert microscopy (Juli, China) for GFP expression. 

***Cell viability assays by micro culture tetrazolium test (MTT assay)***

Cell viability was determined using the MTT assay for transfected SW480 and MDA-MB 231 cells at an initial cell density of 10000 cells per well. This experiment focused on analyzing the ability of expressed metallopeptidase gene derived from *L. casei *to inhibit colorectal cancer cells. 3-(4, 5-Dimethylthiazol-2-yl)-2, 5-diphenyltetrazolium bromide (MTT), a colorless, transparent tetrazolium salt, is reduced to yield a purple formazan crystal through mitochondrial succinate dehydrogenase in living cells. In total, 100 µl (10^5^ cells/ml) of cells was seeded into a 96-well plate, and the cells were cultured overnight at 37 °C in a CO_2_ incubator, which made the cells attach, divide and grow in the 96 wells. The culture medium was removed, 0.2 ml of plasmid and cell culture media without phenol red was added to the respective wells. The cells were incubated for 24 hr. For the analysis, 100 µl of MTT (5 mg/ml) solution was added to each well. After 2 hr culture at 37 °C in a CO_2 _incubator, the supernatants were removed and 100 μl of dimethyl sulfoxide isopropanol was added to dissolve the purple formazan crystals. An ELISA reader (TECAN, Denmark) was used to read the absorbance at 570 nm; next, inhibitory rates were calculated to determine IC50 values. The formula to calculate the inhibitory rate is as follows: Inhibition ratio (%) = [(OD control + OD treated)/ (OD control)] × 100%.

***Determination of apoptosis by TUNEL test***

The apoptosis-inducing effect of metallopeptidase protein on SW480, MDA-MB 231, and blood WBC cells (10^6 ^cells/well) in a 6-well plate was assayed using the TUNEL method with a commercial Dead-End colorimetric TUNEL (Terminal Deoxynucleotidyl Transferase dUTP Nick End Labelling) Kit (Promega, UK) according to the manufacturer’s instructions. Imaging by light microscopy was used for studying and counting the cells.

***The expression analysis of P53 and MAP2K1 genes by real-time RT-PCR***

After 24 and 48 hr from plasmid delivery with three repeats, the cells were harvested and total RNA was extracted from the cells using the Gene All Hybrid-R RNA Purification Kit (Seoul, South Korea) according to the manufacturer’s instructions. The quality and concentration of RNA were examined by ethidium bromide-stained agarose gel electrophoresis and spectrophotometric analysis. Complementary DNA (cDNA) template was synthesized from 1 μg RNA with 100 unit M-MuLv reverse transcriptase enzyme and 2 pmol oligo dT primer (Fermentas, Lithuania). The tubes were incubated at 25 °C for 10 min, 37 °C for 30 min, 42 °C for 60 min, and 70 °C for 5 min. *Actin1* as the housekeeping gene and normalizer for all of the target genes (*P53* and *MAP2K1*) was considered. PCR was used to confirm the quality of the synthesized cDNA. RT-PCR reactions were prepared in a final volume of 13 µl that contained 1 pmol of each primer (Table 1), 1 µg synthesized cDNA, 7 µl DNA Master SYBR Green I mix (Amliqon, Denmark), and 4 µl nuclease-free water. Real-time PCR was performed as follows: 95 °C for 5 min, 95 °C for 10 sec, 60 °C for 15 sec, 72 °C for 20 sec, and 40 cycles of extension. RT-PCR was carried out in a Step One^TM^ instrument (Applied Biosystems, Germany) with *actin 1* as the housekeeping gene.

***Statistical evaluation***

Statistical analysis of the study data was performed using the graphpadprism 8 software package. One-way analysis of variance (ANOVA) or independent sample t-test was used to determine the statistical significance; *P*<0.05 indicates significant difference.

## Results

***Confirmation of the MPL plasmid construct***

**Figure 1:**
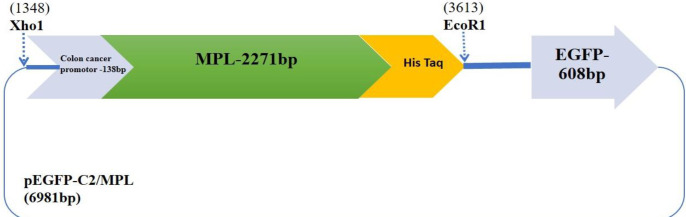
Schematic demonstration of pEGFP-C2/MPL plasmid. Metallopeptidase gene was cloned under the specific promoter in pEGFP-C2 (6984bp)

The synthesized *MPL* gene downstream of the specific colon cancer cell promoter (2271bp) was cloned into a pEGFP-C2 vector (Figure 1). It was confirmed by restriction analysis.

***Colorectal cell-specific promoter performance evaluation by GFP expression***

**Figure 2:**
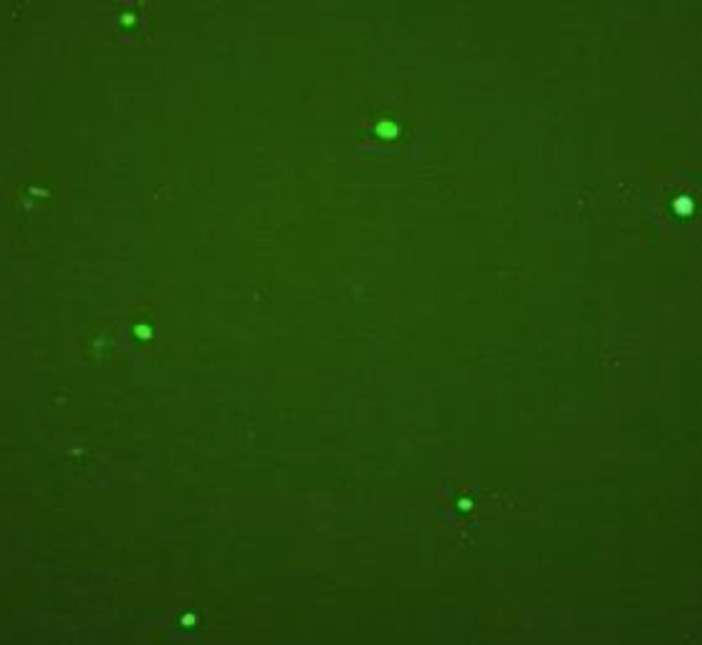
Specific promoter confirmation. GFP expression in SW480 cells 24 hr after transfection with MPL plasmid

Figure 2 shows GFP expression in the cancer cell lines, 24 hr after transfection, and confirms the promoter efficiency that has been inserted before the GFP gene in the plasmid structure (Figure 1). Thereby we expected to express the MPL protein inside the cells.

***Cytotoxic effects of MPL protein***

**Figure 3:**
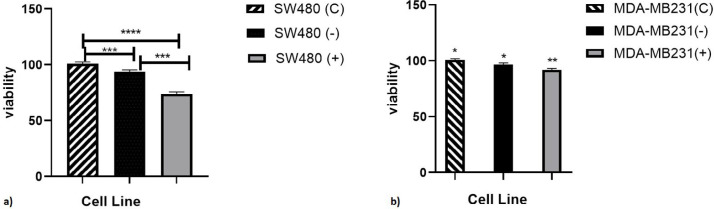
Comparison of cell proliferation between transfected a) SW480 and b) MDA-MB 231 cells. (c) with vector plasmid, (-) without plasmid, (+) with plasmid

With transfection of pEGFP-C2/MPL (MPL gene under colorectal cancer promoter) into SW480 and MDA-MB231 cells, anti-proliferative effect of *L. casei* metallopeptidase protein on cancer cell lines was assayed by cell viability MTT kit. Figure 3 shows the proliferation of these cells in the presence of the endogenous metallopeptidase protein. At the concentration of 10 mg/ml of plasmid, 25% inhibition of SW480 cell proliferation was observed at 48 hr of incubation. *L. casei *metallopeptidase has a lower inhibition effect on the growth of breast cancer cells (7%).

In SW480 negative cells (cells untreated with MPL) the inhibition of growth and proliferation was 8%. In SW480 positive cells (cells treated with MPL) the growth inhibition was 25% in proportion to the control (SW480 cell line treated with pEGFP-C2 plasmid to evaluate the effect of the vector). The results showed that MPL could inhibit the growth and proliferation of SW480 treated cells better than untreated and control cells but in the MDA-MB231 cell line, it was not significant. These results confirm the specific action of the used colorectal cell promoter.

***MPL plasmid promotes apoptotic cell death in colon and breast cancer cells but not in white blood cells as control***

**Figure 4:**
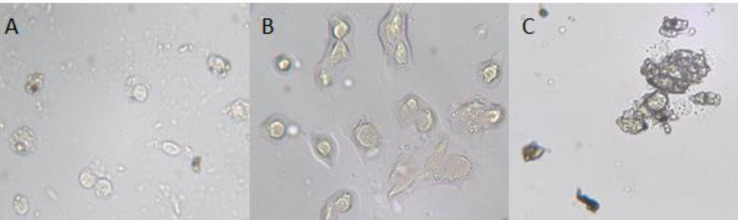
Apoptotic cell assay by DeadEnd Colorimetric TUNEL System. Morphological changes in A: MDA-MB231 cell line, B: Negative Control, C: SW480 cell line, the typical apoptotic cells (dark brown color stained) resulting from nuclear DNA fragmentation

Measuring the effects of this gene on cell death is important in studying the agent’s mechanisms of action. Cell death was monitored by cell imaging using the TUNEL test. We observed signs of cell death in colon cancer cells incubated with the metallopeptidase gene. Delivery of 10 µg plasmid (pEGFP-C2/MPL) to SW480 and MDA-MB 231 cells for 24 hr showed a significant increase in the percentage of both early and late apoptotic cells as compared with healthy blood cells. The blood cells treated with this plasmid (Figure 4B) were healthy, characterized by a round-shape and unstained by TUNEL.

In the SW480 cell line (Figure 4C), a significant number of cells (about 90% of the cell population) stained dark brown and had undergone apoptosis. In MDA-MB 231 cells, the number of apoptotic cells was limited (20%) (Figure 4A). 

***Metallopeptidase of Lactobacillus casei up-regulates expression of the TP53 and MAP2K1 genes in the apoptosis cascade***

**Figure 5:**
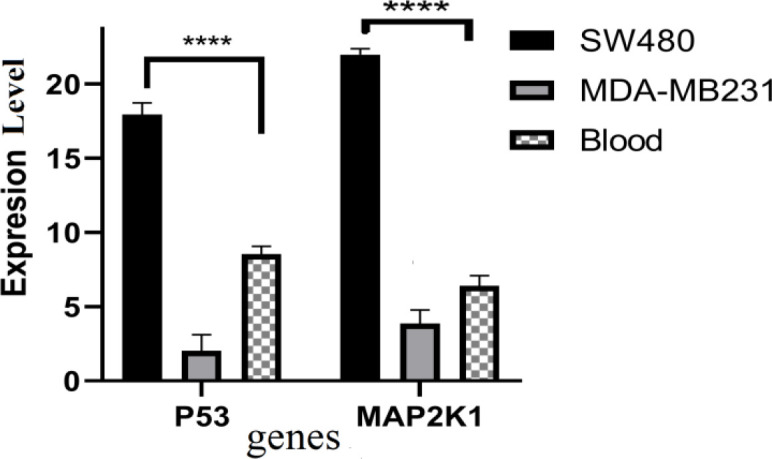
Expression levels of TP53 and MAP2K1 mRNAs at three types of cells after transfection with MPL P<0.05 vs the control group

In order to understand the molecular events leading to apoptosis by MPL expression, we examined the expression of two genes associated with apoptosis. The mRNA expression levels of *TP53* and *MAP2K1* in SW480 and MDA-MB 231 cell lines were measured by RT-qPCR. Following transfection, mRNA expression levels of *TP53* and *MAP2K1* were up-regulated compared with the control after 48 hr (Figure 5). 

**Table1:** Primers used in real-time RT-PCR of apoptosis pathway genes

**Fragment length (bp)**	**Primer sequence (5** **′- ** **3** **′)**	**Gene**
215	AGCACTAAGCGAGCACTG	*TP53* F
	CTGGGCATCCTTGAGTTCC	*TP53* R
250	GCGGAGACCAACTTGGAGCG	*MAP2K1* F
	CATCCTTCAGTTCTCCCACC	*MAP2K1* R
200	TGTTGGAGTGGATCCGCCGCACAA	*ACTN1* F
	CATCCTGCCCTCAGAGGGCATGAA	*ACTN1* R

TP53: tumor protein p53; MAP2K1: mitogen-activated protein kinase; ACTN1: actinin alpha1; F: Forward; R: Reverse

## Discussion

All of the traditional cancer therapies, including various surgeries, hormonal therapies, immune therapies, etc., show a lack of efficacy in terms of the long-term outcome because of their failure to target cancer cells and toxicity due to non-specific effects on normal cells (26). Probiotics and gut commensals can modulate the host’s gut epithelial barrier function via their surface molecules and metabolites (2, 10). According to several studies (9, 27-29) *L. casei* ATCC 39392 as a key probiotic microorganism exerts beneficial health-promoting activities including immunomodulatory, anti-inflammatory, and anti-tumor properties. The common compounds produced by the *Lactobacillus* strains are lactic acid, exopolysaccharides, biosurfactants, and peptidases that have anti-cancer effects and play important roles in maintenance and regulation of cell functions (30). Our understanding of the biological processes involved in the beneficial effects of LAB strains is still limited. In particular, the role of probiotic bacteria’s structural and secretory proteins in producing beneficial effects has been little studied. For this purpose, the present study evaluates the effects of an acid-resistant recombinant protein identified in the *L. casei *ATCC 39392 strain against growth inhibition of colon and breast cancers in a cell culture model. 

In our previous study an ATP-dependent metallopeptidase (FtsH/Yme1/Tma family protein), from the lactic acid bacterium *L. casei *was identified by proteomics approaches (25). The metallopeptidase gene encodes a protein of 714 amino acid residues, which displays at least 90% identity with this protein in *Lactobacillus* genera. Its expression is induced after decreased pH from 7 to 5. This protein plays an important role in the physiology and survival of bacteria in intestinal acid conditions. 

Thus, a gene fragment encoding metallopeptidase protein of *L. casei* ATCC 39392 under specific colon cancer cell promoter was considered and the anti-proliferative and apoptotic effects of the endogenous expressed metallopeptidase protein were evaluated on SW480 and MDA-MB231 cell lines and compared with WBC blood cells as control. Numerous studies have been done on the effect of this bacterium on cancers, including Tiptiri-Kourpeti’s research on the growth-inhibitory effects of *L. casei* ATCC 39392 against experimental colon cancer. He found administration of live *L. casei * (as well as bacterial components thereof) on murine (CT26) and human (HT29) colon carcinoma cell lines raised a significant concentration and time-dependent antiproliferative effect; live *L. casei* induced apoptotic cell death in both cell lines (9). Therefore, more detailed research on each of the proteins of this bacterium is necessary to obtain its effective protein and design drugs effective against cancer.

More work is needed in order to reveal the causative underlying characteristics responsible for specific antitumor effects. Survey effects of metallopeptidase isolated from *Lactobacillus* on SW480, MDA-MB231 showed that metallopeptidase induces *p53* expression in SW480 cells and apoptosis occurs in the cells treated with this protein more than MDA-MB231 cells.

The application from tissue-specific promoters is one of the ways for cancer cells targeted therapy. According to Ghanbariasad *et al*. mammaglobin-1 expression is restricted to the mammary glands and no expression has been reported in various types of benign tissue or neoplasia other than breast carcinoma. They introduced the mammaglobin-1 promoter as a cancer specific promoter with high efficacy (31). Thus, colon cancer specific promoter may limit the damage to healthy cells by expressed MPL protein.

Also, for evaluation of the specificity of the promoter function, we study the cytotoxicity and growth inhibition of the endogenous metallopeptidase as a protein derived from *Lactobacillus* on human colon and breast cancer cell lines. MPL gene construct with specific promoter was found to inhibit the growth of colon cancer cells significantly as detected by the MTT assay. But it had no effect on the growth inhibition of the breast cancer cell line. This gene has the potential to inhibit the proliferation of SW480 cells.

In this regard, Kim *et al*. also investigated the effect of extracts of *L. casei* ATCC 39392 on human cervical cell lines Caski and HeLa, and asserted that the extract did not affect the growth of women’s cancer cell lines, and there was no synergistic effect after concomitant administration of one or more chemotherapeutic drugs. Thus, *L. casei *extract may have an anti-cancer effect in cervical cancer through an effect on cell cycle arrest, although it has no effect on growth inhibition or any synergistic effect on women’s cancer cell growth when administered together with a chemotherapeutic drug (32). Many studies have reported about the beneficial effects of metabolites of this genus of bacteria, for example, Chuah *et al*. showed that postbiotic metabolites (PM) produced by the six strains of *L. plantarum* exhibited selective cytotoxicity via antiproliferative effect and induction of apoptosis against malignant cancer cells in a strain-specific and cancer cell type-specific manner whilst sparing the normal cells (33). Thus, due to the differences between the two cancer cells in our study, the mechanisms and pathways of apoptosis induced under the treatment with the metallopeptidase encoded plasmid in the two cell types, did not demonstrate the same. Another research demonstrated that poly P, a cytoprotective compound from *Lactobacillus brevis* SBC8803 is the molecule responsible for maintaining intestinal barrier actions which are mediated through the intestinal integrin b1-p38 MAPK (34). These results indicate that metallopeptidase produced from the acidic metabolism in *L. casei *is most likely involved in the probiotic effects described for this bacterium. *TP53* is a gene that interferes with cell cycle activities and is a tumor suppressor gene. When a tumor suppressor gene is mutated, cell proliferation is uncontrolled (35,36). Disruption of *TP53* tumor suppressor gene regulation is one of the most common events in CRC stimulation, and gene reactivation may be a good suggestion for treatment of colon cancer. P53 can activate mitochondrial apoptosis pathways during cell stress. MAP2K1 is uniquely linked to *TP53* mutation. *In silico* interaction between *P53* and *MAP2K1* has been shown (37). Therefore, a cell with a *P53* mutated allele will lose its natural tendency to regulate programmed cell death and may survive due to genotoxic factors. Mutation in *p53* is the most frequently detected genetic alteration in human cancers (38). Increased expression of this tumor suppressor protein leads to inhibition of tumor cell growth and apoptosis. Proteins isolated from probiotics lead to transcriptional activation of genes whose expression inhibits tumor growth. MAP2K1 is a member of the large family of Ser/Thr kinases, which triggers multiple rounds of hierarchical phosphorylation-activating kinase circles from the cell surface to the nucleus (39, 40-42). Also, mitogen-activated protein kinase (MAP kinase) is a key signal-transducing protein that transmits signals involved in both cell proliferation and apoptosis (43-45). In some articles have reported expression and clinical significance of MAPK in breast cancer (40, 46-49). Studies have examined the effect of different strains of *Lactobacillus *on breast cancer (50) and CRC (51).

Therefore, due to the important role of MAP2K1 and P53 proteins in preventing apoptosis, we decided to investigate the effect of endogenous protein expression of MPL with an intestinal cell specific promoter on expression of MAP2K1 and P53 in MPL protein treated human colorectal and breast cancer cell lines, and human normal white blood cells as a control. So, we proposed that this protein (metallopeptidase) inhibits cancer cell proliferation that is related to the modulation of apoptotic signaling-regulated proteins.

## Conclusion

Transfer of plasmid-containing gene MPL under specific intestinal promoter by protein endogenous expression is able to induce apoptosis in colorectal cancer cells. This promoter acts specific and the MPL gene is not expressed in healthy and non-intestinal cells and does not cause harm.

## References

[1] Kahouli I, Tomaro-Duchesneau C, Prakash S (2013). Probiotics in colorectal cancer (CRC) with emphasis on mechanisms of action and current perspectives. J Med Microbiol.

[2] Irecta-Najera CA, Huizar-Lopez MR, Casas-Solis J, Castro-Felix P, SanterreA (2017). Protective effect of Lactobacillus casei on DMH-induced colon carcinogenesis in mice. Probiotics & Antimicro Prot.

[3] Dorostkar R, Hashemzadeh MS, Jafari S, Tat M, Ghalavand M, Asghari MH (2016). Immunotherapeutic efficacy of a Lactobacillus casei lysate as an adjuvant combined with a heated-4T1mammary carcinoma cell lysate in a murine model of breast cancer. Asian Biomed.

[4] Globocan Estimated cancer incidence, mortality and prevalence Worldwide in 2012.

[5] An BC, Hong S, Park HJ, Kim BK, Ahn JY, Ryu Y (2019). Anti-colorectal cancer effects of probiotic-derived p8 protein. Genes.

[6] Bauerl C, Perez-Martinez G, Yan F, Polk DB, Monedero V (2010). functional analysis of the p40 and p75 proteins from Lactobacillus casei BL23. J Mol Microbiol Biotechnol.

[7] Lin J, Zou Y, Ma C, Liang Y, Ge X, Chen Z (2016). Construction and characterization of three protein-targeting expression system in Lactobacillus casei. FEMS Microbiol Lett.

[8] Claes IJJ, Schoofs G, Regulski K, Courtin P, Chapot-Chartier M-P, Rolain T (2012). Genetic and biochemical characterization of the cell wall hydrolase activity of the major secreted protein of Lactobacillus rhamnosus GG. PLoS One.

[9] Tiptiri-Kourpeti A, Spyridopoulou K, Santarmaki V, Aindelis G, Tompoulidou E, Lamprianidou EE (2016). Lactobacillus casei exerts anti-proliferative effects accompanied by apoptotic cell death and up-regulation of TRAIL in colon carcinoma cells. PLoS ONE.

[10] Liu Q, Yu Z, Tian F, Zhao J, Zhang H, Zhai Q (2020). Surface components and metabolites of probiotics for regulation of intestinal epithelial barrier. Microb Cell Fact.

[11] Sanchez B, Urdaci M C, Margolles A (2010). Extracellular proteins secreted by probiotic bacteria as mediators of effects that promote mucosa–bacteria interactions. Microbiol.

[12] Wang Y, Liu L, Moore DJ, Shen X, Peek RM, Acra SA (2017). An LGG-derived protein promotes IgA production through upregulation of APRIL expression in intestinal epithelial cells. Mucosal Immunol.

[13] Siciliano RA, Mazzeo MF (2012). Molecular mechanisms of probiotic action: a proteomic perspective. Curr Opin Microbiol.

[14] Kumar M, Nagpal R, Verma V, Kumar A, Kaur N, Hemalatha R (2013). Probiotic metabolites as epigenetic targets in the prevention of colon cancer. Nutr Rev.

[15] Thomas CM, Versalovic J (2010). Probiotics-host communication: Modulation of signaling pathways in the intestine. Gut Microbes.

[16] Soltan Dallal MM, Mojarrad M, Baghbani F, Raoofian R, Mardaneh J, Salehipour Z (2015). Effects of probiotic Lactobacillus acidophilus and Lactobacillu casei on colorectal tumor cells activity (CaCo-2). Arch Iran Med.

[17] Han DJ, Kim JB, Park SY, Yang MG, Kim H (2013). Growth Inhibition of Hepatocellular Carcinoma Huh7 Cells by Lactobacillus casei Extract. Yonsei Med J.

[18] Cicenia A, Scirocco A, Carabotti M, Pallotta L, Marignani M, Severi C (2014). Postbiotic activities of Lactobacilli-derived factors. J Clin Gastroenterol.

[19] Savabi O, Kazemi M, Kamali S, Salehi AR, Eslami G, Tahmourespour A (2014). Effects of biosurfactant produced by Lactobacillus casei on gtfB, gtfC, and ftf gene expression level in S. mutans by real-time RT-PCR. Adv Biomed Res.

[20] Mbye M, Baig MA, AbuQamar SF, El-Tarabily KA, Obaid RS, Osaili TM (2020). Updates on understanding of probiotic lactic acid bacteria responses to environmental stresses and highlights on proteomic analyses. Compr Rev Food Sci Food Saf.

[21] Adu KT, Wilson R, Nichols DS, Baker AL, Bowman JP, Britz ML (2018). Proteomic analysis of Lactobacillus casei GCRL163 cell-free extracts reveals a SecB homolog and other biomarkers of prolonged heat stress. PLoS One.

[22] Hosseini Nezhad M, Hussain MA, Britz ML (2015). Stress responses in probiotic Lactobacillus casei. Crit Rev Food Sci Nutr.

[23] Papadimitriou K, Alegria A, Bron PA, De Angelis M, Gobbetti M, Kleerebezem M (2016). Stress physiology of lactic acid bacteria. Microbiol Mol Biol Rev.

[24] Wu R, Sun Z, Wu J, Meng H, Zhang H (2010). Effect of bile salts stress on protein synthesis of Lactobacillus casei Zhang revealed by 2-dimensional gel electrophoresis. J Dairy Sci.

[25] Dadfarma N, Karimi G, Nowroozi J, Nejadi N, Kazemi B, Bandehpour M (2020). The Proteomic Analysis of Lactobacillus casei in Response to Different pHs Using Two-Dimensional Electrophoresis and MALDI TOF Mass Spectroscopy. Iran J Microbiol.

[26] Sadhu A, Ganguly K (2017). K. Lactobacillus sp. A Threat to Pathogenic Microorganisms and Tumor Cells. J Cancer Ther.

[27] Xu C, Qiao L, Guo Y, Ma L, Cheng Y (2018). Preparation, characteristics and antioxidant activity of polysaccharides and proteins-capped selenium nanoparticles synthesized by Lactobacillus casei 393. Carbohydr Polym.

[28] Kavousipour S, Khademi F, Zamani M, Vakili B, Mokarram P (2017). Novel biotechnology approaches in colorectal cancer diagnosis and therapy. Biotechnol Lett.

[29] Sidira M, Galanis A, Ypsilantis P, Karapetsas A, Progaki Z, Simopoulos C (2010). Effect of Probiotic-Fermented Milk Administration on Gastrointestinal Survival of Lactobacillus casei ATCC 393 and Modulation of Intestinal Microbial Flora. J Mol Microbiol Biotechnol.

[30] Maftuni K, Zare P (2017). Effects of Lactobacillus casei culture supernatant on differentiation of K562 cell line. Med Lab J.

[31] Ghanbariasad A, Bandehpour M, Yaghoobi H, Kazemi B (2018). Suicide gene therapy for breast cancer with a suicide-inducing vector carrying the mammaglobin-1 promoter. Minerva Biotechnol.

[32] Kim SN, Lee WM, Park KS, Kim JB, Han DJ, Bae J The effect of Lactobacillus casei extract on cervical cancer cell lines. Contemp Oncol (Pozn)2015.

[33] Chuah LO, Foo HL, Loh TC, Mohammed Alitheen NB, Yeap SK, Abdul Mutalib NE (2019). Postbiotic metabolites produced by Lactobacillus plantarum strains exert selective cytotoxicity effects on cancer cells. BMC Complement Altern Med.

[34] Segawa S, Fujiya M, Konishi H, Ueno N, Kobayashi N, Shigyo T (2011). Probiotic-derived polyphosphate enhances the epithelial barrier function and maintains intestinal homeostasis through integrin–p38 MAPK pathway. PLoS One.

[35] Jerjees DA, AL-Irhayim BA (2009). P53 expression in colonic carcinoma immunohistochemical study. Ann Coll Med.

[36] Azarhoush R, Heidari K, Samadzadeh S, Heidari, Mehravar F (2018). Expression of p53 Protein in Colorectal Cancer and Association with Prognostic Factors in Northeast Iran. Acta Med Iran.

[37] Rostami-Nejad M, Rezaei Tavirani S, Mansouri V, Jahani Sherafat S, Moravvej Farshi H (2019). Gene expression profile analysis of colon cancer grade II into grade III transition by using system biology. Gastroenterol Hepatol Bed Bench.

[38] Ghavam-Nasiri MR, Rezaei E, Ghafarzadegan K, Seilanian-Toosi M, Malekifard H (2007). Expression of p53 in Colorectal Carcinoma: Correlation with Clinicopathologic Features. Arch Iran Med.

[39] Koveitypour Z, Panahi F, Vakilian M, Peymani M, Forootan FS, Nasr Esfahani MH (2019). Signaling pathways involved in colorectal cancer progression. Cell Biosci.

[40] Yang XL, Liu KY, Lin FJ, Shi HM, Ou ZL (2017). CCL28 promotes breast cancer growth and metastasis through MAPK-mediated cellular anti-apoptosis and pro-metastasis. Oncol Rep.

[41] Guo YJ, Pan WW, Liu SB, Shen ZF, Xu Y, Hu LL (2020). ERK/MAPK signaling pathway and tumorigenesis. Exp Ther Med.

[42] Low HB, Zhang Y (2016). Regulatory Roles of MAPK Phosphatases in Cancer. Immune Netw.

[43] Santen R J, Song RX, McPherson R, Kumar R, Adam L, Jeng MH (2002). The role of mitogen-activated protein (MAP) kinase in breast cancer. J Steroid Biochem Mol Biol.

[44] Dai C, Zhao DH, Jiang M (2012). VSL3 probiotics regulate the intestinal epithelial barrier in vivo and in vitro via the p38 and ERK signaling pathways. Int J Mol Med.

[45] Jiang W, Wang X, Zhang C, Xue L, Yang L (2020). Expression and clinical significance of MAPK and EGFR in triple negative breast cancer. Oncol Lett.

[46] Li X, Wang K, Ren Y, Zhang L, Tang XJ, Zhang HM (2014). MAPK signaling mediates sinomenine hydrochlorideinduced human breast cancer cell death via both reactive oxygen species-dependent and -independent pathways: an in vitro and in vivo study. Cell Death Dis.

[47] Liu Z, Ren L, Liu C, Xia T, Zha X, Wang S (2015). Phenformin Induces Cell Cycle Change, Apoptosis, and Mesenchymal-Epithelial Transition and Regulates the AMPK/mTOR/p70s6k and MAPK/ERK Pathways in Breast Cancer Cells. PLoS One.

[48] Jiang L, Wang Y, Liu G, Liu H, Zhu F, Ji H (2018). C Phycocyanin exerts anti cancer effects via the MAPK signaling pathway in MDA MB 231 cells. Cancer Cell Int.

[49] Lee S, Rauch J, Kolch W (2020). Targeting MAPK Signaling in Cancer: Mechanisms of Drug Resistance and Sensitivity. Int J Mol Sci.

[50] Kaga C, Takagi A, Kano M, Kado S, Kato I, Sakai M (2013). Lactobacillus casei Shirota enhances the preventive efficacy of soymilk in chemically induced breast cancer. Cancer Sci.

[51] Yanagihara S, Fukuda S, Ohno H, Yamamoto N (2012). Exposure to Probiotic Lactobacillus acidophilus L-92 Modulates Gene Expression Profiles of Epithelial Caco-2 Cells. J Med Food.

